# Supplementary figures and images for: Modelling chemotaxis of branched cells in complex environments provides insights into immune cell navigation
Source: PLoS Comput Biol. 2026 Feb 3;22(2):e1013934. doi: 10.1371/journal.pcbi.1013934 (PMC12880755; doi:10.1371/journal.pcbi.1013934)

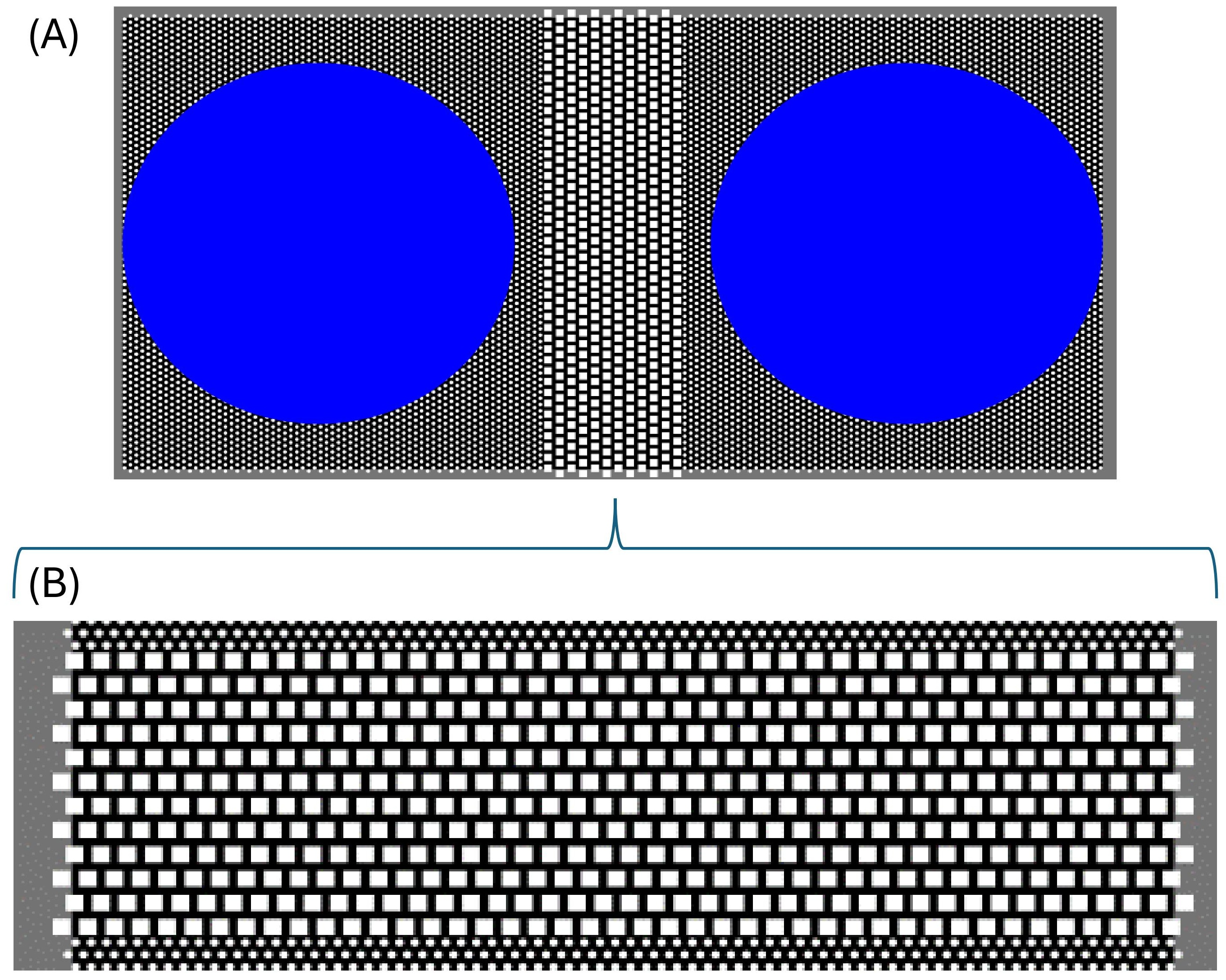

Supplement: S1 Fig — (A) Blue dots indicate loading areas for cells and chemoattractant. Loading areas are surrounded by an area of circular pillars, which serve as an antechamber from where cells enter the 0.7x2.5 mm analysis area. (B) Analysis area harbors 10x10 μm rectangular pillars of 3.8 μm height. Pillars are distanced 5 μm and 3 μm in vertical vs. horizontal direction, and arranged in a hexagonal lattice. (TIFF) [file pcbi.1013934.s001.tiff]

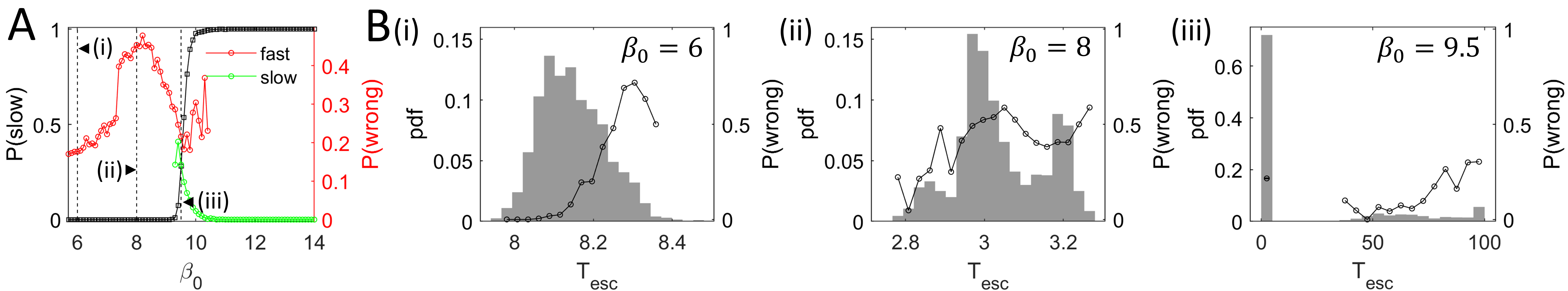

Supplement: S2 Fig — (A) Mean probability of entering the slow mode at the junction, P(slow) (black line), and the error rates of the fast and slow processes (red and green lines, respectively). (B) (i–iii) Distributions of escape time Tesc (histograms) and error probability P(wrong) (black line) as functions of Tesc, for the β0 values indicated by the vertical dashed lines in (A). Key parameters: ϵ=0.001, σ=0.1. (TIFF) [file pcbi.1013934.s002.tiff]

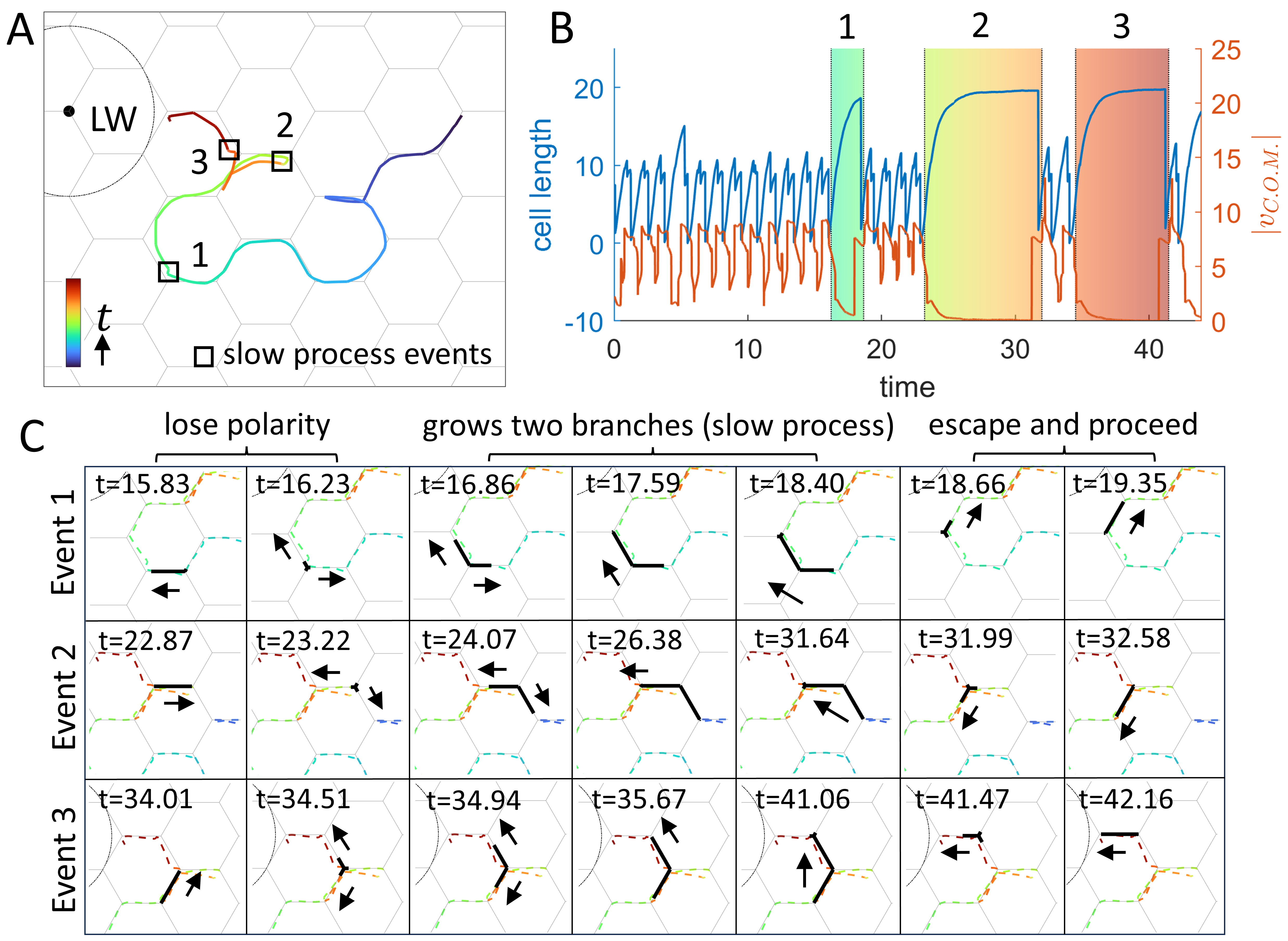

Supplement: S3 Fig — (A) Representative simulation trajectory of a cell migrating toward the LW, with trajectory color denoting migration time. Black boxes highlight time intervals along the trajectory where slow mode events occur, corresponding to the colored regions in (B) and the snapshots in (C). (B) Dynamics of cell length and C.O.M. speed during migration. The three colored regions correspond to the slow mode events marked in (A). (C) Simulation snapshots of the cell during the slow mode events. Parameters: C/c0=0.01,ϵ=0.2,d=9,β0=12,σ=1.2. (TIFF) [file pcbi.1013934.s003.tiff]

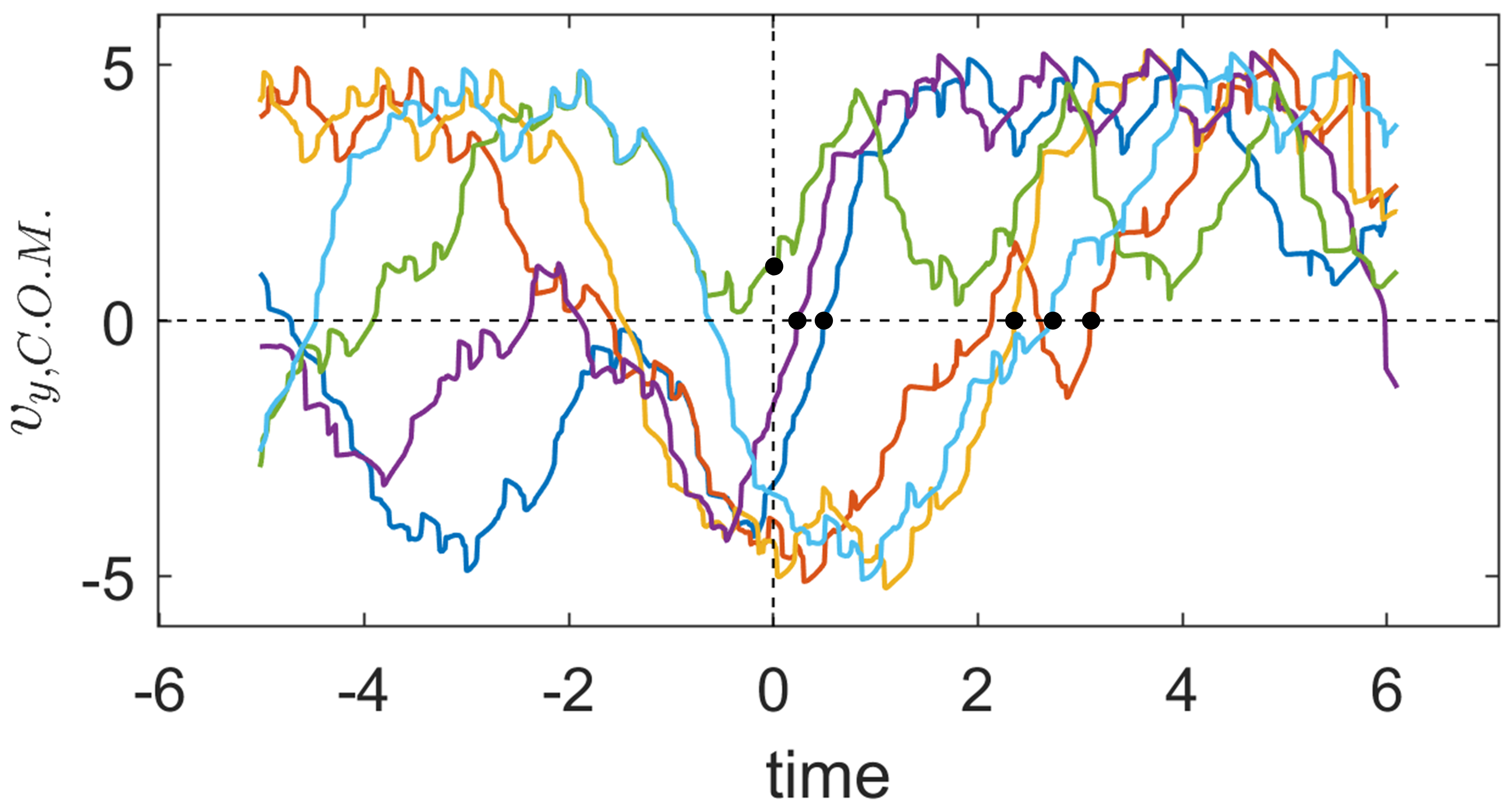

Supplement: S4 Fig — Time evolution of vy,C.O.M. for six randomly selected simulation trajectories. Black dots mark the BM time for each trajectory, i.e., the moment the cell first begins to move toward the source (positive y-direction). (TIFF) [file pcbi.1013934.s004.tiff]

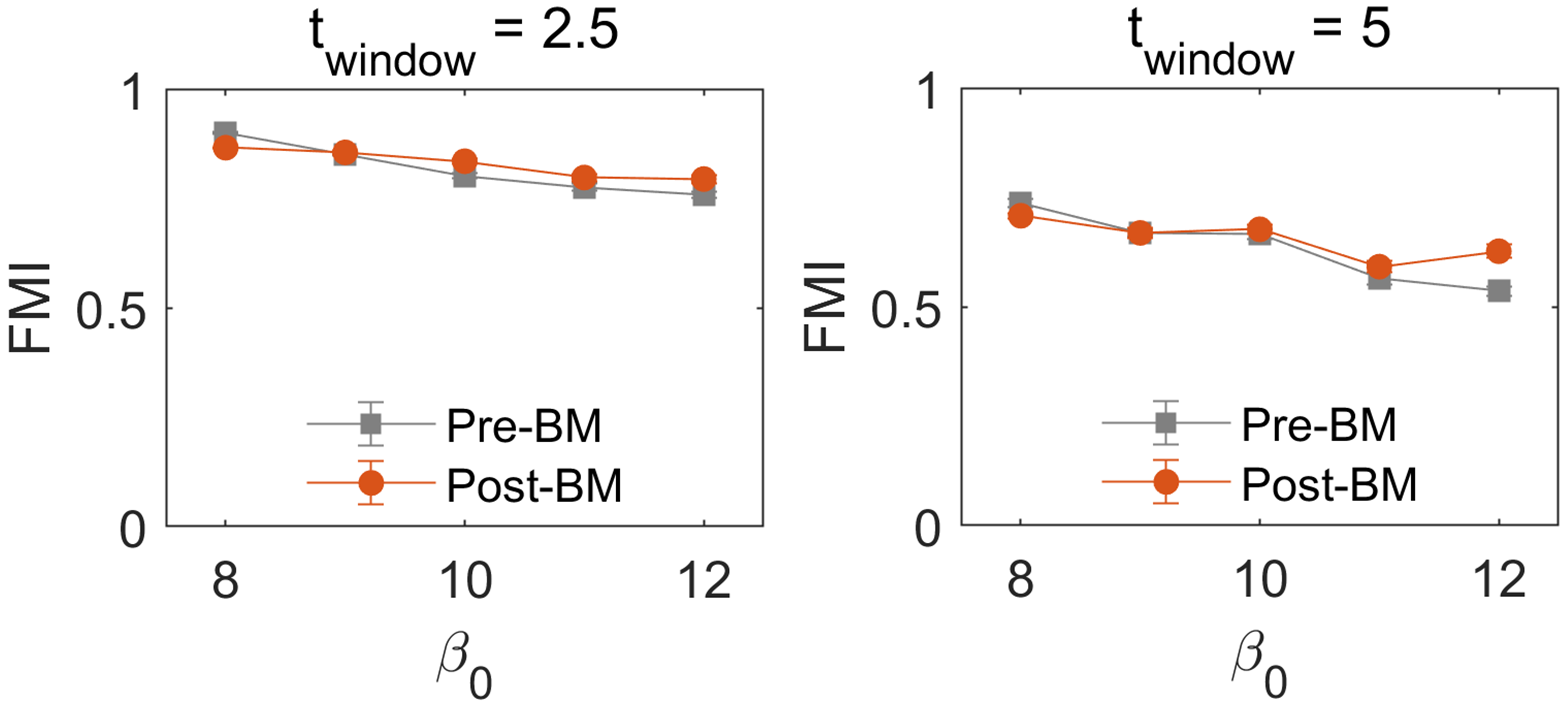

Supplement: S5 Fig — Gray and red correspond to the FMI before and after the BM time, respectively. Key parameters: ϵ=0.1,d=3,σ=0.5. (TIFF) [file pcbi.1013934.s005.tiff]

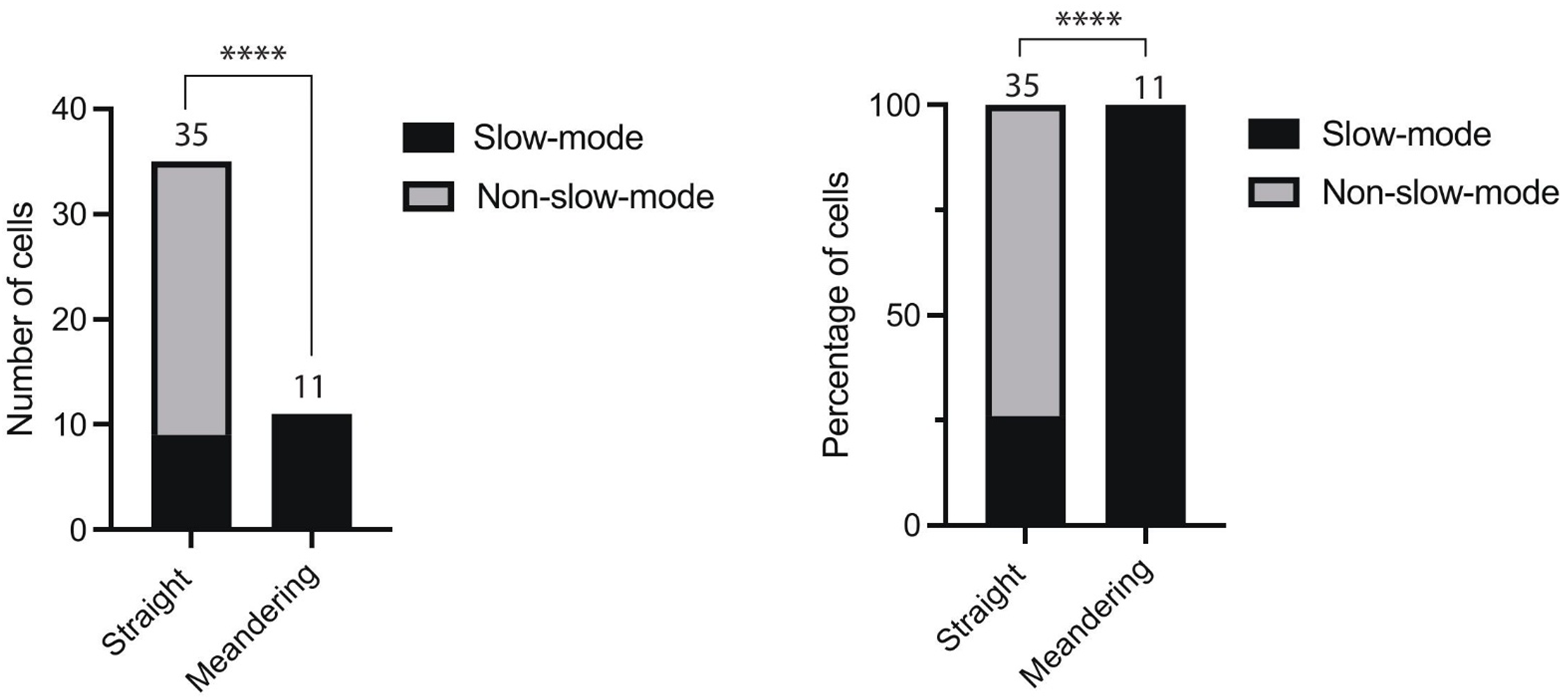

Supplement: S6 Fig — GCamp6F transgenic zebrafish embryos (3 dpf) were infected with Pseudomonas aeruginosa, laser-wounded, and imaged using a two-photon confocal microscope, as previously described. Neutrophil trajectories were manually tracked using the IMARIS software. Trajectories were classified as meandering or straight based on the FMI quantified by the software (>0.60 considered straight). They were further categorized by the presence or absence of slow mode events. The graphs show both the absolute counts (left) and percentages (right) of cells displaying slow mode events within the straight and meandering categories. Percentages are based on 39 and 11 cells, respectively, as indicated. Videos with overall recruitment of more than 15 cells were selected. In total, N = 46 neutrophil trajectories from 7 independent videos/embryos were analyzed. ****P<0.0001, Chi-square test (and Fisher’s exact test). (TIFF) [file pcbi.1013934.s006.tiff]
