# Supplementary material for: Modelling chemotaxis of branched cells in complex environments provides insights into immune cell navigation
Source: PLoS Comput Biol. 2026 Feb 3;22(2):e1013934. doi: 10.1371/journal.pcbi.1013934 (PMC12880755; doi:10.1371/journal.pcbi.1013934)
Supplement: S1 Appendix — (PDF) [file pcbi.1013934.s007.pdf]

S1 Appendix. Calculation of the steady-state distribution of the polarity cue concentration and the local actin treadmilling flows

The polarity cue concentration along arm i, $c_i(x_i)$, is obtained as the steady-state solution of the diffusion–advection equation describing the polarity cue within each cell segment [1, 2]

$$\frac{\partial}{\partial x}(u_i\, c_i(x) + D\,\frac{\partial c_i(x)}{\partial x}) = 0 \tag{S-1}$$

where the net actin flow in that segment, u_i, is given by Eq. 7, and D is the diffusion coefficient of the polarity cue.

Applying a no-flux boundary condition at the distal end of each arm,

$$u_i c_i(x) + D\frac{\partial c_i(x)}{\partial x}\bigg|_{x=x_i} = 0 \tag{S-2}$$

the steady-state solution to Eq. 17 takes the exponential form

$$c_i(x) = c_{i,0}\ \exp\left(-\frac{u_i x}{D}\right) \tag{S-3}$$

The relationships among the coefficients $c_{i,0}$ are determined by imposing the continuity of the polarity cue concentration at the junction nodes. For instance, for a cell spanning two junctions, let the polarity cue concentration at junction P be denoted by c_0 (Fig. A). Then, the concentration at the adjacent junction Q is given by $c_0\ \exp\left(-\frac{u_m d}{D}\right)$, where u_m is the net actin flow in the middle segment of the cell, defined as positive from Q to P. Accordingly, the concentration profiles along all cell segments can be written as

$$c_m(x_m) = c_0\ \exp\left(-\tfrac{u_m\, x_m}{D}\right) \tag{S-4}$$

$$c_{1/2}(x_{1/2}) = c_0\ \exp\left(-\tfrac{u_{1/2}\, x_{1/2}}{D}\right) \tag{S-5}$$

$$c_{3/4}(x_{3/4}) = c_0\ \exp\left(-\tfrac{u_m d}{D}\right)\ \exp\left(-\tfrac{u_{3/4}\, x_{3/4}}{D}\right) \tag{S-6}$$

as shown in Fig. A.

The total amount of the polarity cue within the cell is conserved and given by

$$c_{tot} = \sum_i \int_0^{x_i} c_i(x_i)\ dx_i \tag{S-7}$$

where the summation runs over all cell segments.

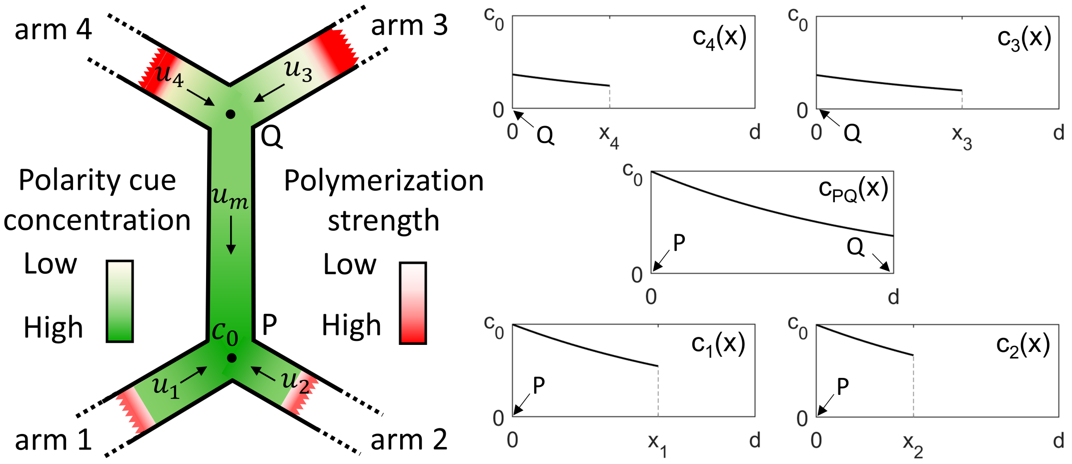

Fig. A: Illustration of the spatial distribution of the polarity cue concentration. Left: Schematic representation of the polarity cue concentration field (green scale), advected by the actin flows (black arrows), together with the corresponding actin polymerization strength (red scale). Right: Steady-state polarity cue concentration profiles along all cell segments.

For the case of a cell spanning two junctions, this becomes

$$c_{tot} = c_0 D \left[\frac{1-\exp\left(-\frac{u_m d}{D}\right)}{u_m} + \frac{1-\exp\left(-\frac{u_1 x_1}{D}\right)}{u_1} + \frac{1-\exp\left(-\frac{u_2 x_2}{D}\right)}{u_2} + \exp\left(-\frac{u_m d}{D}\right)\left(\frac{1-\exp\left(-\frac{u_3 x_3}{D}\right)}{u_3} + \frac{1-\exp\left(-\frac{u_4 x_4}{D}\right)}{u_4}\right)\right] \tag{S-8}$$

From this expression, c_0 can be expressed in terms of c_{tot}, D, d, x_i, and u_i. Substituting back into Eq. 20, the concentration profiles c_i can likewise be expressed as functions of these parameters.

In general, one can arbitrarily select a junction node as the reference point O, assign its concentration as c_0, compute the total concentration c_{tot}, and subsequently express both c_0 and $c_i(x_i)$ in terms of c_{tot}, D, d, x_i, and u_i. The general expression of $c_i(x_i)$ is given by [2]

$$c_i(x) = \frac{c_{tot}}{D} \frac{\exp\left(-\frac{\sum_{k_{Oi}} u_{k_{Oi}} d}{D}\right)\exp\left(-\frac{u_i x}{D}\right)}{\sum_l \exp\left(-\frac{\sum_{k_{Ol}} u_{k_{Ol}} d}{D}\right)\frac{1-\exp\left(-\frac{u_l d}{D}\right)}{u_l} + \sum_j \exp\left(-\frac{\sum_{k_{Oj}} u_{k_{Oj}} d}{D}\right)\frac{1-\exp\left(-\frac{u_j x_j}{D}\right)}{u_j}} \tag{S-9}$$

In this expression, the term $\sum_{k_{Oi}}$ in the numerator denotes the cumulative contribution of the node-connecting segments between the reference node O and arm i; each segment corresponds to a link between two adjacent junctions that does not include local actin flows. In the denominator, l represents the node-connecting segments of the cell, and for each l, the summation $\sum_{k_{Ol}}$ includes all segments between O and segment l. Similarly, j represents the free arms of the cell, and the summation $\sum_{k_{Oj}}$ includes all node-connecting segments between O and arm j.

The steady-state actin treadmilling flow at the tip of arm i is given by

$$v_i^* = \beta \frac{c_s}{c_s + c_i(x_i)} = \beta \frac{1}{1 + \frac{c_i(x_i)}{c_s}} \tag{S-10}$$

where c_s is the dissociation constant of the polarity cue.

By defining the normalized polarity cue concentration as $\widetilde{c}_i(x_i) = \frac{c_i(x_i)}{c_s}$, we obtain Eq. 6, where

$$\widetilde{c}_i(x) = \frac{c}{D} \frac{\exp\left(-\frac{\sum_{k_{Oi}} u_{k_{Oi}} d}{D}\right)\exp\left(-\frac{u_i x}{D}\right)}{\sum_l \exp\left(-\frac{\sum_{k_{Ol}} u_{k_{Ol}} d}{D}\right)\frac{1-\exp\left(-\frac{u_l d}{D}\right)}{u_l} + \sum_j \exp\left(-\frac{\sum_{k_{Oj}} u_{k_{Oj}} d}{D}\right)\frac{1-\exp\left(-\frac{u_j x_j}{D}\right)}{u_j}} \tag{S-11}$$

and $c = \frac{c_{tot}}{c_s}$ is the ratio between the total polarity cue concentration and its dissociation constant.

By combining Eqs. 6, 7 and S-11, we obtain a system of implicit equations for the variables v_i. Numerical solutions of this system yield the values of v_i corresponding to a given set of x_i.

Table A: Model parameter values used in this study. All lengths and times in the model are normalized by characteristic experimental length and time scales [1]: Time is normalized by the inverse focal-adhesion disassembly timescale and length by the cell's rest length scale on one-dimensional tracks [3], so all parameters are dimensionless.

Parameter	Description	Value	First Eq.
k	Effective cell elasticity	0.8	Eq. 1
r	Cell–substrate adhesiveness	5	Eq. 2
f_s	Susceptibility of the adhesion linker to the applied force	5	Eq. 2
δ	Response rate of the local actin flow	250	Eq. 3
κ	Adhesion linker elasticity	20	Eq. 4
D	Diffusion coefficient of the polarity cue	3.85	Eq. S-1
c	Total concentration / dissociation constant of the polarity cue	3.85	Eq. S-11

[1] J. E. Ron, M. Crestani, J. M. Kux, J. Liu, N. Al-Dam, P. Monzo, N. C. Gauthier, P. J. Sáez, and N. S. Gov, Nature Physics pp. 1–11 (2024).

[2] J. Liu, J. Boix-Campos, J. E. Ron, J. M. Kux, N. S. Gov, and P. J. Sáez, arXiv preprint arXiv:2404.00118 (2024).

[3] J. E. Ron, P. Monzo, N. C. Gauthier, R. Voituriez, and N. S. Gov, Physical Review Research **2**, 033237 (2020).
