# Supplementary material for: Modelling chemotaxis of branched cells in complex environments provides insights into immune cell navigation
Source: PLoS Comput Biol. 2026 Feb 3;22(2):e1013934. doi: 10.1371/journal.pcbi.1013934 (PMC12880755; doi:10.1371/journal.pcbi.1013934)
Supplement: S2 Appendix — (PDF) [file pcbi.1013934.s008.pdf]

S2 Appendix. Onset of the stick-slip motion for cells on a one-dimensional line

Stick–slip migration arises naturally in our model, as previously shown in [1]. For the same parameters as in Fig. 3B(i), we demonstrate the onset of stick–slip motion on a one-dimensional line once β exceeds the threshold value $\beta^{1D}_{stick-slip} \simeq 8.95$ (Fig. S-1, vertical blue line in Fig. 3B(i)). The threshold for stick-slip is lower for cells that are just arriving at a junction compared to lotion on an infinite line, as shown in Fig. 3B(i).

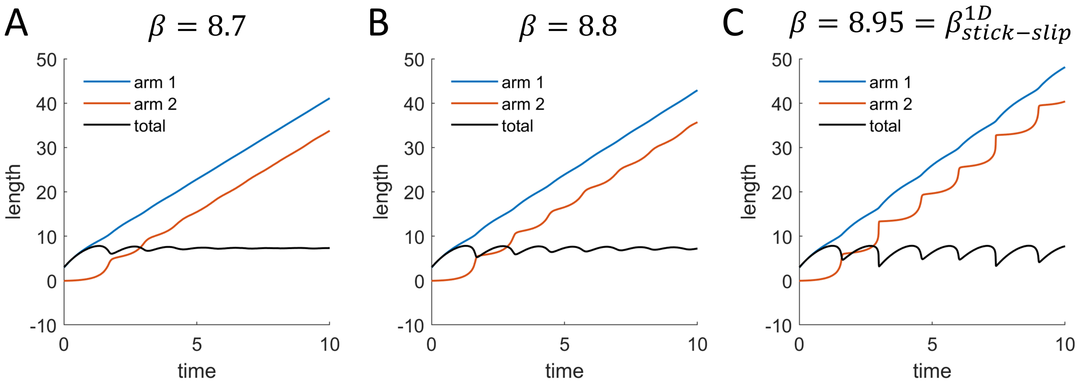

Fig. S-1: Onset of the stick-slip motion for cells on a one-dimensional line. (A), (B) and (C) show examples for $\beta = 8.7$, $\beta = 8.8$, and $\beta = 8.95$, respectively. The critical value of β for stable stick-slip motion is $\beta^{1D}_{stick-slip} = 8.95$.

[1] J. E. Ron, P. Monzo, N. C. Gauthier, R. Voituriez, and N. S. Gov, Physical Review Research **2**, 033237 (2020).
