# Supplementary material for: Modelling chemotaxis of branched cells in complex environments provides insights into immune cell navigation
Source: PLoS Comput Biol. 2026 Feb 3;22(2):e1013934. doi: 10.1371/journal.pcbi.1013934 (PMC12880755; doi:10.1371/journal.pcbi.1013934)
Supplement: S3 Appendix — (PDF) [file pcbi.1013934.s009.pdf]

S3 Appendix. Effect of cellular internal noise and grid size on chemotaxis dynamics

We investigated how cellular internal noise affects chemotactic migration. For both the strong and weak-signal regimes (Fig. S-1A,B, respectively), increasing noise leads to larger $\langle T_{\rm arr}\rangle$ and $\langle L_{\rm path}\rangle$, along with smaller $\langle v_y\rangle$ and $\langle FMI\rangle$, indicating reduced migration efficiency.

In the weak-signal regime (Fig. S-1B), $\langle L_{\rm path}\rangle$ saturates for $\beta_0 > 8$, suggesting there is no further decrease in accuracy. This is consistent with the decreasing $\langle T_{\rm arr}\rangle$ and increasing $\langle v_y\rangle$ in this range of β_0. In Fig. S-1C we plot the probability of arrival within $T_{\max}$, P(arrival), as a function of β_0 for the weak-signal regime. For large noise,

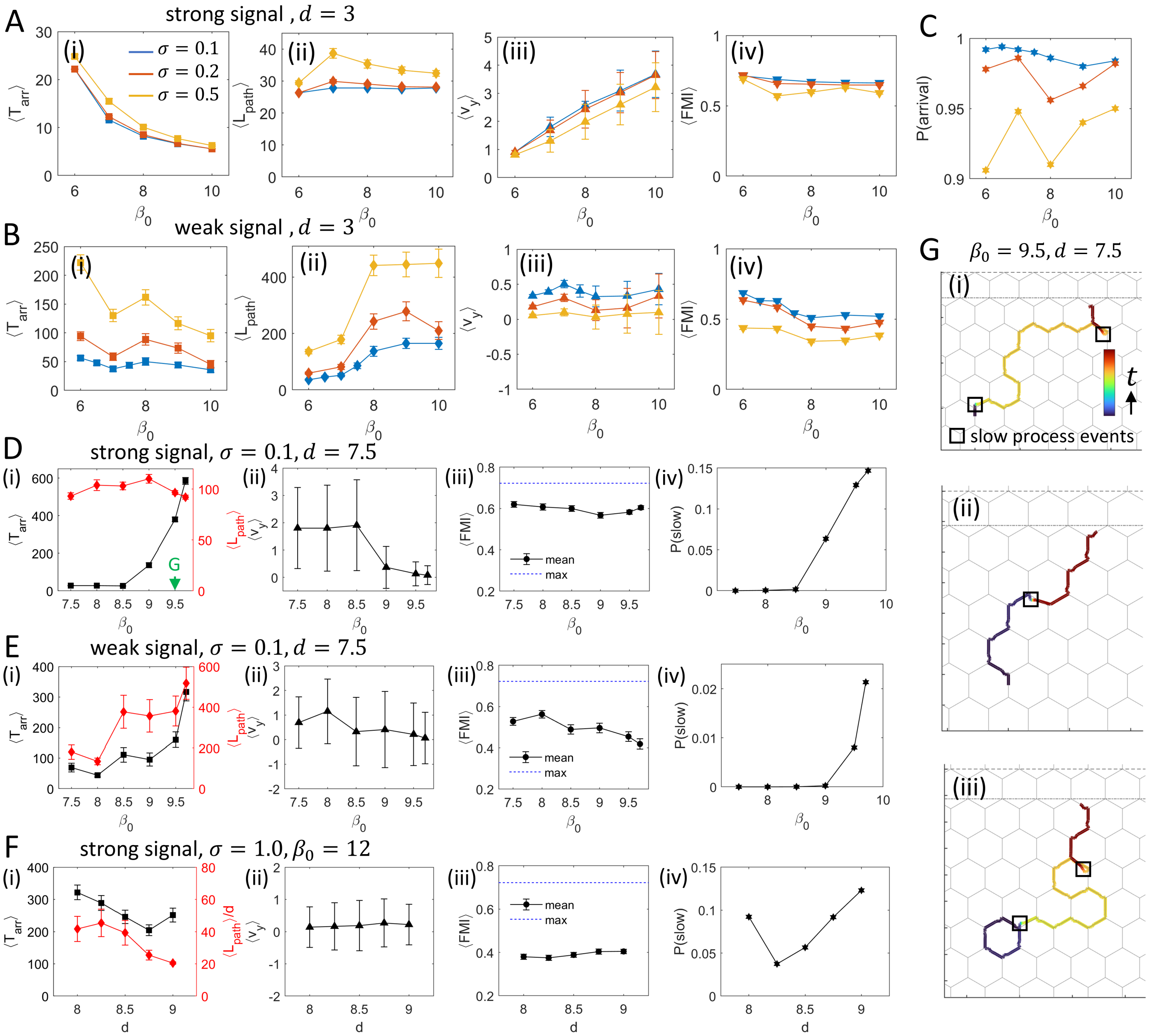

Fig. S-1: Effect of cellular internal noise σ and grid size d on chemotaxis dynamics. (A–B) Small grids ($d = 3$): (i) $\langle T_{\rm arr}\rangle$, (ii) $\langle L_{\rm path}\rangle$, (iii) $\langle v_y\rangle$, and (iv) $\langle FMI\rangle$ as functions of β_0. (A) and (B) correspond to the strong and weak-signal regimes, respectively. (C) Probability of arrival at the source at $T_{\max} = 1000$ for the weak-signal case in (B). (D–E) Large grids ($d = 7.5$): (i) $\langle T_{\rm arr}\rangle$ and $\langle L_{\rm path}\rangle$, (ii) $\langle v_y\rangle$, (iii) $\langle FMI\rangle$ (black solid line) and the theoretical maximum FMI (blue dashed line), and (iv) P(slow) as functions of β_0. (D) and (E) correspond to the strong and weak-signal regimes, respectively. (F) Effect of grid size d on (i) $\langle T_{\rm arr}\rangle$ and $\langle L_{\rm path}\rangle$, (ii) $\langle v_y\rangle$, (iii) $\langle FMI\rangle$, and (iv) P(slow) for $\beta_0 = 12$, in the strong-signal regime (as in (A)) under high noise ($\sigma = 1.0$). (G) Representative trajectories of the cell's C.O.M. for $\beta_0 = 9.5$, $d = 7.5$, and $\sigma = 0.1$. Black squares indicate slow-mode events along the trajectory. Maximal simulation time: $T_{\max} = 1000$. Other key parameter: $\epsilon = 0.2$.

P(arrival) peaks coincide with the minima of $\langle T_{\rm arr}\rangle$ in Fig. S-1B(i), with a distinct minimum around $\beta_0 \sim 8$.

We also examined the migration dynamics on larger grids (Fig. S-1D-F). Cells with high β_0 frequently exhibited slow-mode events, with their probability P(slow) increasing sharply as β_0 rose (Fig. S-1D,E(iv)), leading to a marked increase in $T_{\rm arr}$ (Fig. S-1D,E(i)). Representative trajectories for the parameters indicated by the green arrow in Fig. S-1D(i) are shown in Fig. S-1G, with slow-mode events along the paths marked by black squares. For the strong-signal regime, these slow events slightly enhance migration accuracy, as reflected by the trends in $\langle L_{\rm path}\rangle$ and $\langle FMI\rangle$ (Fig. S-1D(i,iii)). This effect, however, is not observed under weak-signal conditions (Fig. S-1E(i,iii)).

Finally, in Fig. S-1F, we present the dependence of migration dynamics on grid size d under strong-signal conditions, with large $\beta_0 = 12$ and high noise level $\sigma = 1$. Increasing d leads to higher $P_{\rm slow}$ and improved accuracy, as indicated by the rise in $\langle FMI\rangle$ and the reduction in $\langle L_{\rm path}\rangle$.
