# Supplementary material for: Modelling chemotaxis of branched cells in complex environments provides insights into immune cell navigation
Source: PLoS Comput Biol. 2026 Feb 3;22(2):e1013934. doi: 10.1371/journal.pcbi.1013934 (PMC12880755; doi:10.1371/journal.pcbi.1013934)
Supplement: S4 Appendix — (PDF) [file pcbi.1013934.s010.pdf]

S4 Appendix. Detailed analysis of cell trajectory dynamics in the weak-signal regime

For the weak-signal regime and $\beta_0 = 8$, we plot the distributions of $T_{\rm arr}$ and $L_{\rm path}$ (Fig. S-1A). Both distributions exhibit discrete peaks with constant spacing. Insets of (A) display the distributions with probability on a logarithmic scale, highlighting their approximately exponential form. By examining the trajectories corresponding to the first six peaks of $T_{\rm arr}$ and $L_{\rm path}$ (Fig. S-1B), we conclude that these regular intervals arise from the extra time and path length accumulated whenever the cell makes an additional incorrect decision (away from the chemokine source).

We next plot the distributions of y and v_y (Fig. S-1C(i–ii)), whose discrete peaks reflect the periodic migration of the cell along the hexagonal edges. The mean $\langle v_y \rangle$ (Fig. S-1C(iii)) and the v_y distributions within selected y-ranges (Fig. S-1C(iv)) show that $\langle v_y \rangle$ becomes positive only at sufficiently large y, where the cell can sense the chemokine source, whereas farther away it loses the weak signal and undergoes isotropic diffusion.

To illustrate this behavior, Fig. S-1D,E present examples of efficient and inefficient migration paths, corresponding to the 1st and 15th peaks in Fig. S-1A. Panels (i)–(iii) show the trajectories, the corresponding v_y distributions, and the v_y time series, where shaded regions mark the ranges defined in (ii). The periodic v_y dynamics indicate stick–slip migration over the hexagonal network. Panel (iv) further shows representative snapshots of the cell shape, C.O.M., and v_y vectors at the time points marked in (iii).

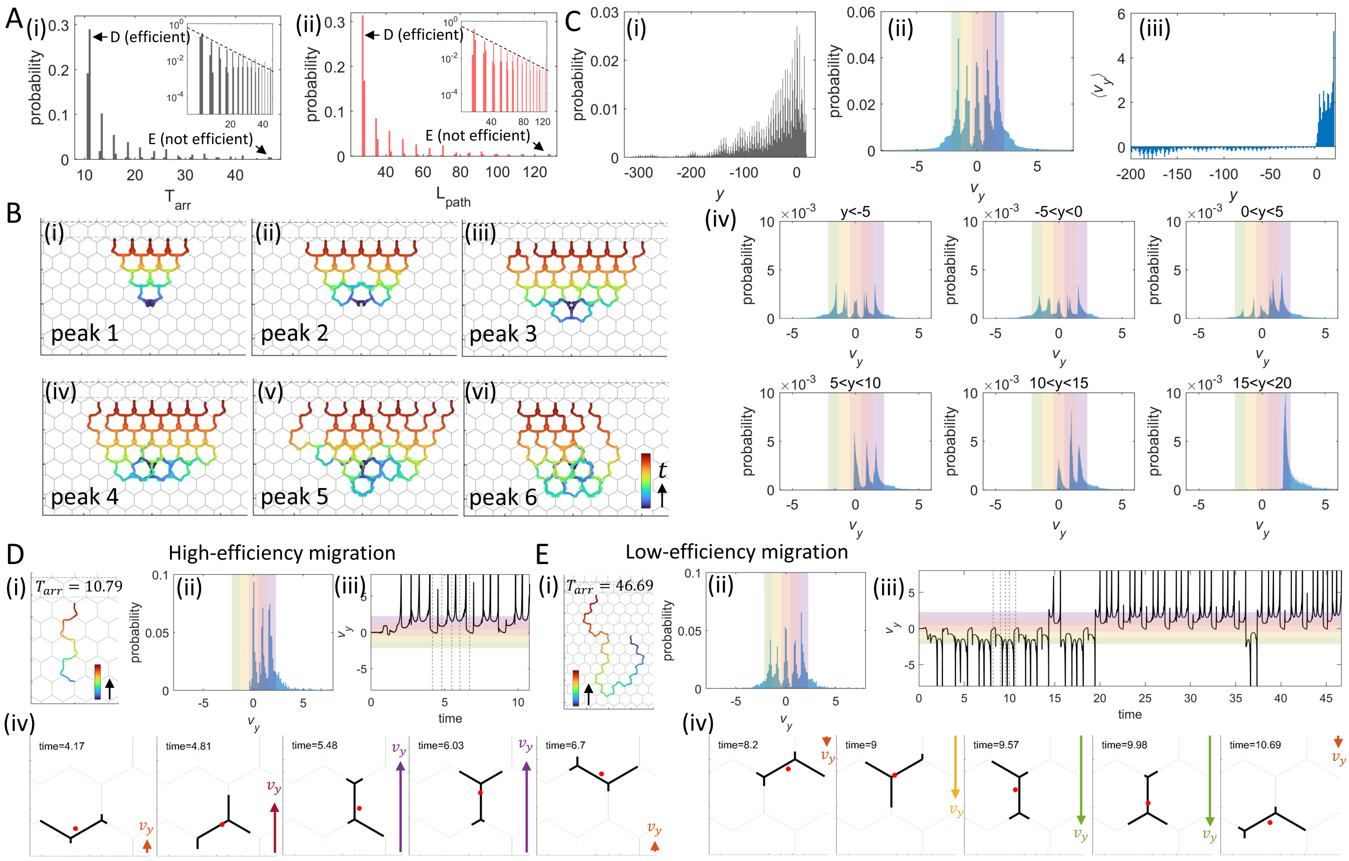

Fig. S-1: Detailed analysis of cell trajectory dynamics in the weak-signal regime. (A) Distributions of (i) T_{arr} and (ii) L_{path}. Insets show the same distributions with a logarithmic y-axis, revealing their approximately exponential tails. (B) Trajectories corresponding to the first six peaks of the T_{arr} and L_{path} distributions in (A). (C) Distributions of (i) the y-position and (ii) v_y of the cell's C.O.M.; (iii) v_y distributions within different y-ranges; (iv) mean $\langle v_y \rangle$ as a function of y. Colored shaded areas in (ii) and (iv) denote different v_y ranges, consistent with the colored shading in (D) and (E). (D–E) Examples of efficient and inefficient migration. (i) C.O.M. trajectory; (ii) distribution of v_y during the trajectory; (iii) time series of v_y, with gray dashed lines marking the time points shown in (iv); (iv) snapshots of cell shape corresponding to these time points. Colored arrows denote the v_y vectors corresponding to the shaded regions of the same color. (D) and (E) correspond to efficient and inefficient migration, respectively. Key parameters: $\epsilon = 0.2, d = 3, \beta_0 = 8, \sigma = 0.1$.
