# Supplementary material for: Modelling chemotaxis of branched cells in complex environments provides insights into immune cell navigation
Source: PLoS Comput Biol. 2026 Feb 3;22(2):e1013934. doi: 10.1371/journal.pcbi.1013934 (PMC12880755; doi:10.1371/journal.pcbi.1013934)
Supplement: S5 Appendix — (PDF) [file pcbi.1013934.s011.pdf]

S5 Appendix. Comparing the experimental response of the cell to the chemokine with the model

In comparing the model with the experiments, we assumed a linearly decaying chemokine line source (Fig. S-1, left). The concentration is oriented along the y-axis, reaching its maximum value c_0 at the source position y_{source} and dropping to zero at y_{end}. Between these positions, the chemokine concentration varies linearly, such that the function $c(y)$ in our model (Eq. 16) takes the form

$$c(y) = c_0 \frac{y - y_{\text{end}}}{y_{\text{source}} - y_{\text{end}}}. \tag{S-1}$$

We set $y_{\text{source}} = -y_{\text{end}} = 8d$. At the start of each simulation, the cell is symmetrically positioned at the origin $(x, y) = (0, 0)$. The chemokine profile parameters were set to $\epsilon = 0.1$ and $C/c_0 = 1$.

Under these conditions, the normalized chemokine concentration and the enhancement of actin activity as functions of y are shown in Fig. S-1, right panel. Although the chosen parameter $C/c_0 = 1$ corresponds to the weak-signal regime for the exponentially decaying chemokine source, the linear variation of $c(y)$ ensures that once the chemical cue is introduced, the cell still experiences a substantial signal.

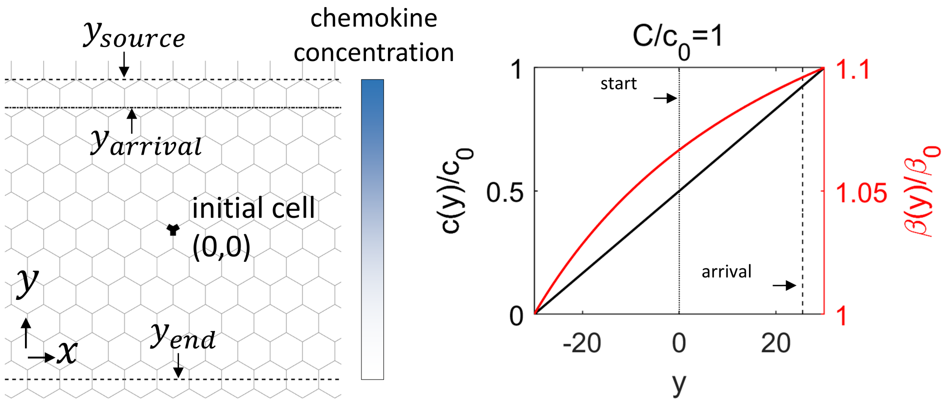

Fig. S-1: Model illustration with a linearly decaying chemokine line source. Left: Schematic of the model. Two gray dashed lines mark the position of the chemokine source and the position where the source decays to zero, respectively. The dash-dotted lines marks the arrival position. Right: Normalized chemokine concentration, $c(y)/c_0$ (black line), and the enhancement of actin activity, $\beta(y)/\beta_0$, as functions of y.
