# Supplementary material for: Modelling chemotaxis of branched cells in complex environments provides insights into immune cell navigation
Source: PLoS Comput Biol. 2026 Feb 3;22(2):e1013934. doi: 10.1371/journal.pcbi.1013934 (PMC12880755; doi:10.1371/journal.pcbi.1013934)
Supplement: S6 Appendix — (PDF) [file pcbi.1013934.s012.pdf]

S6 Appendix. Analysis of the cell's response to the chemokine gradient in experiments and simulations

In Fig. S-1A,B(i), we show the dynamics of the cell's C.O.M. velocity in the y-direction, $|v_{y,\text{C.O.M.}}|$, and the angle θ between the C.O.M. velocity vector and the positive y-axis for both the experiment and the simulation (see schematic in Fig. 5A,B). In both cases, cells slow down following the introduction of the chemokine gradient, then repolarize and accelerate toward the source. In this process, the migration angle exhibits oscillations of similar magnitude in both cases.

The origin of this deceleration in the model can be understood by examining the maximal front–back difference in the polarity cue along the cell length (Fig. S-1B(ii)) and the polarity concentrations at the arm tips (Fig. 5B). Before the chemokine gradient is introduced, the polarity cue difference oscillates as the cell periodically changes its length and number of arms spanning the junction while migrating on the hexagonal network. At time $t = 0$, the cell is polarized such that its leading edge points away from the source, exposing its rear to higher chemokine concentrations than its front. Consequently, actin polymerization is transiently stronger at the cell rear than at the front. A similar transient enhancement of actin activity on the side facing the chemokine gradient is also observed in neutrophils [1]. This imbalance reduces the overall front–back actin treadmilling flow and weakens advection of the polarity cue. As a result, the front–back polarity cue difference decreases (Fig. S-1B(ii)), slowing cell migration. Reduced polarity makes protrusive activity more uniformly distributed across all protrusions, facilitating cell rotation. Once front–back polarity is re-established in the direction of the gradient (the polarity cue difference regains large values, Fig. S-1B(ii)), the cell resumes rapid migration.

In Fig. S-1B(iii), we show the dynamics of the arm tips with the largest (front) and smallest (back) y-coordinates. The cell's length along the y-axis is plotted in Fig. S-1B(iv), showing that the cell elongates in the gradient direction. Finally, the chemokine-induced enhancement of actin activity at the front and back tips is shown in Fig. S-1B(v). Their difference, plotted in Fig. S-1B(vi), decreases gradually over time as the cell migrates up the gradient and the overall chemokine concentration surrounding the cell increases.

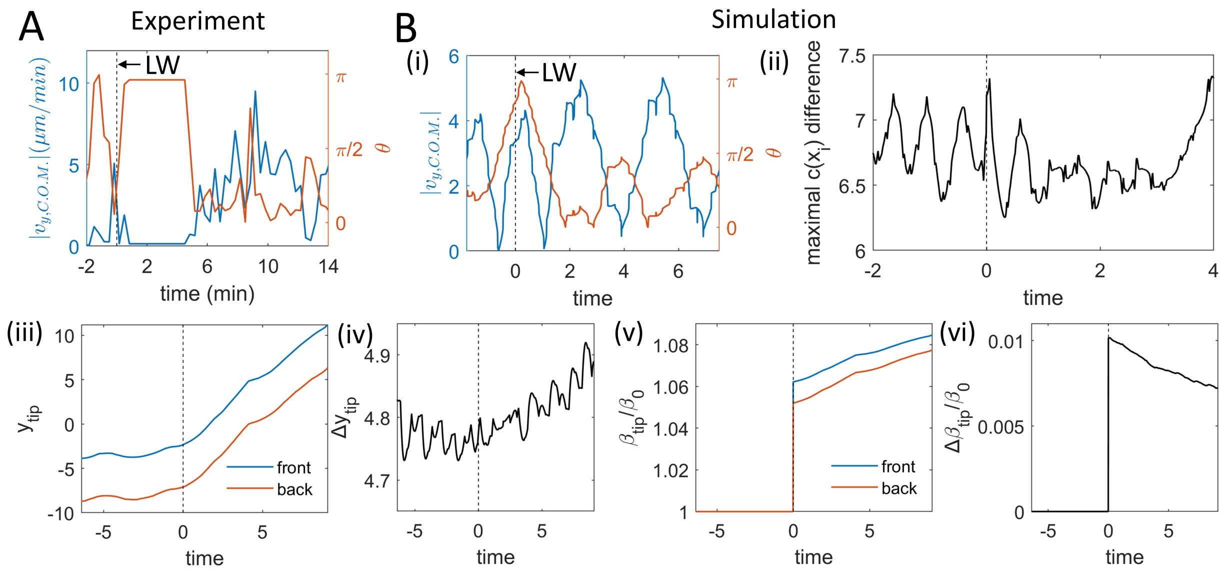

Fig. S-1: Chemotaxis dynamics of cells on a hexagonal network with a linearly decaying chemokine line source. (A) Dynamics of the C.O.M. velocity in y-direction, $|v_{y,\text{C.O.M.}}|$, of the experimental cell (main text Fig. 5). (B) Dynamics of the simulated cell's (i) C.O.M. velocity in the y-direction, $|v_{y,\text{C.O.M.}}|$, (ii) maximal difference between the polarity cue concentration at the arm tips, $c(x_i)$, (iii) y-coordinates of the front tip (maximum y-coordinate) and back tip (minimum y-coordinate), (iv) difference in y-coordinates between the front and back tips, (v) β enhancement, β/β_0 at the front and back tips, (vi) difference in β enhancement between the front and back tips. Black dashed lines mark the LW time. Parameters: $\epsilon = 0.1, d = 3, \beta_0 = 12, \sigma = 0.5$.

[1] A. Georgantzoglou, H. Poplimont, H. A. Walker, T. Lämmermann, and M. Sarris, Journal of Cell Biology **221**, e202103207 (2022).
