# Supplementary material for: Modelling chemotaxis of branched cells in complex environments provides insights into immune cell navigation
Source: PLoS Comput Biol. 2026 Feb 3;22(2):e1013934. doi: 10.1371/journal.pcbi.1013934 (PMC12880755; doi:10.1371/journal.pcbi.1013934)
Supplement: S7 Appendix — (PDF) [file pcbi.1013934.s013.pdf]

S7 Appendix. Comparison of the chemotaxis characteristics of drug-treated cells between simulations and neutrophil experiments

The total cell length is observed to be significantly diminished by decreasing actin activity (CK666, Fig. S-1A)[1]. Compared to the WT cells, we fit a reduced β_0, by $\sim 17\%$ in the CK666-affected cells (Fig. S-1B). The effect of blebbistatin, which inhibits myosin-II contractility, is described in our model as a decrease in the cell contractile-stiffness parameter k and the actin activity β_0. While decreasing k acts to make the cell longer, the lower β_0 decreases the forces that act to elongate the cell, so that combined the cell is similar in length to the WT, as observed in the experiments (Fig. S-1A,B)[1].

In Fig. S-1C,D, we compare the experimental [1] and simulated changes to the FMI for the WT (DMSO) and drug-treated cells. The relative changes are qualitatively captures by the model, especially the increase in FMI due to the chemokine for the WT cells, and the vanishing of this effect upon drug application. This, again, points to

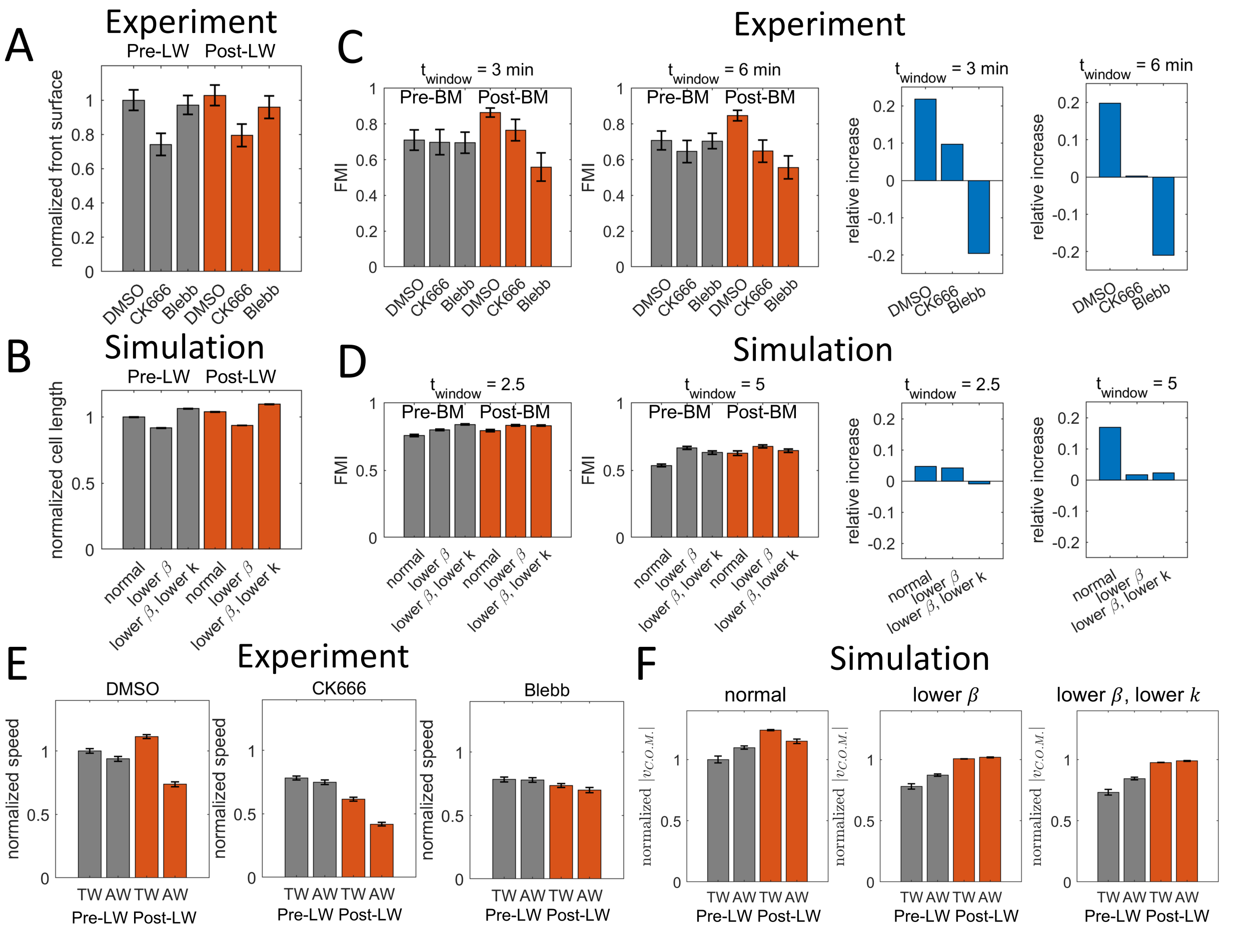

Fig. S-1: Comparison of the chemotaxis characteristics of drug-treated cells between simulations and neutrophil experiments [1]. (A) Normalized front surface of experimental cells (normalized by the DMSO value before LW), before and after the LW event (gray and red, respectively). (B) Normalized cell length of simulated cells (normalized by the normal cell value before LW), before and after the LW event (gray and red, respectively). (C) FMI of the experimental cell's C.O.M. under different drug treatments within a specific time window (left two panels) and the relative increase after BM compared to before BM (right two panels). (D) FMI of the simulated cell's C.O.M. under different parameter settings (corresponding to the respective drug treatments in experiments) within a specific time window (left two panels) and the relative increase after BM compared to before BM (right two panels). (E) Normalized speed of the experimental cell's C.O.M. (normalized by the DMSO value in TW(pre)), toward (TW) and away (AW) from the wound, before and after the LW event (gray and red, respectively). (F) Normalized speed of the simulated cell's C.O.M. (normalized by the untreated value in TW(pre)), before and after BM (gray and red, respectively). For WT (normal) cells, CK666-treated cells, and blebbistatin-treated cells, the corresponding simulation parameters are set to $(\beta, k) = (12.0, 0.8)$, $(\beta, k) = (10.0, 0.8)$, and $(\beta, k) = (10.0, 0.7)$, respectively. Other key parameters: $\epsilon = 0.1, d = 3, \sigma = 0.5$.

the WT cells residing in the high-β_0 regime of our model, which is the only regime where we see that the FMI is significantly increased by the chemokine gradient.

Similarly, in Fig. S-1E,F the normalized cell speed is compared between the WT and drug-treated cells [1]. In the WT cells, a large difference is found between the cell speed towards and against the chemokine direction, while this is very much reduced for the drug-treated cells, which also have lower overall migration speed compared to the WT (Fig. S-1E). These qualitative features are captured by the model (Fig. S-1F).

[1] A. Georgantzoglou, H. Poplimont, H. A. Walker, T. Lämmermann, and M. Sarris, Journal of Cell Biology **221**, e202103207 (2022).
