# Supplementary material for: Modelling chemotaxis of branched cells in complex environments provides insights into immune cell navigation
Source: PLoS Comput Biol. 2026 Feb 3;22(2):e1013934. doi: 10.1371/journal.pcbi.1013934 (PMC12880755; doi:10.1371/journal.pcbi.1013934)
Supplement: Attachment — Submitted filename: Reviewers_response letter.docx [file pcbi.1013934.s015.docx]

**Reviewers' comments:**

Reviewer's Responses to Questions

**Reviewer #1:** The authors investigate the motion of neutrophils in a "complex" environment (in vivo in zebrafish, in vitro with pillars, and simulated in hexagonal arrays of micro-patterns). They compare experimental data to a model of the "branching cells" that is basically a set of spring-like arms competing for dominance. The eventual takeaway is that there is a a speed-accuracy trade-off.

The authors show simulations for a single cell at a junction point and later for a single cell in a hexagonal array. They compare various model predictions to the behaviour of real neutrophils in the experiments.

- We thank the reviewer for an overall positive and constructive report. Indeed part of our take-home message is the existence of a speed-accuracy trade-off, but this effect is not predicted to be strong under realistic conditions. The second take-home message is the conclusion, based on comparison to experiments, that neutrophils are residing in the high-speed-low-accuracy regime.

Major issues

Overall, while the title and abstract were very enticing, the paper as a whole was, at its current state, less interesting than expected. Perhaps the authors assumed that everyone is familiar with their (previously published) model, and so spent little attention on motivating the assumptions and assessing both benefits and shortcomings. Perhaps the take-away speed-accuracy trade-off seems unsurprising. Or perhaps, the overall presentation could have used more thoughtful self-critical analysis and better discussion, to avoid a reader's reaction of "so what?". It is overall not a reader-friendly presentation. Probably, the authors could improve the writing to make this into the interesting paper that it deserves to be.

- We agree with the reviewer that the manuscript was not optimally written, and we have worked to improve the clarity, as well as explain and emphasize the main results more clearly.

The description of the model in the main text is cursory, and anticipates that the reader will be familiar with refs [18, 26]. The main assumptions should be here (not only in the SI) and justified as key properties that are necessary and sufficient for describing neutrophils.

- We followed the advice of the reviewer and added a full description of the model and its basic assumptions and ingredients in the main text, under the section “Materials and methods, Theoretical model”, which we moved from the Appendix.

Review of work on cells in mazes, cells creating or adapting the local gradients, and other models for cell arms based on competing springs was not very complete.

- We have added several relevant references to the Introduction section, and throughout the paper.

The authors' discussion of tests of the model are summarized in a quick statement on L413-415, and in Fig S7.
"We incorporate the inhibition of actin polymerization or myosin-II activity in our model through changes to the model parameters, as was done previously [18]."
The values, of parameters, but not a justification, is given in Fig S7. The assumed effects of the various inhibitors is not explained. Again, the paper should be self-contained.

- The explanation for the choice of parameter changes in our model that represent the effects of the different drugs used in the experiment, is given in Appendix S7 (previous Appendix S5). We failed to direct the reader to this section in the relevant place in the text, and this is now corrected.

In general, there are a great many graphs and bar charts, but the overall message is hard to parse. The figures are hard to digest because they have series that go horizontally as well as vertically in the same figure, the caption are very terse, and there is a lot of notation to memorize. The abbreviations and notation make it hard to get the message of what the authors intend to convey. A short conclusion or significance statement in the captions could be helpful.

- We followed the advice of the reviewer and simplified the labels of the panels in Fig.3 (previous Fig.2) of the main text. We also added more explanatory text to the captions, to convey the message of each figure more clearly.

Figure S1 seems to be missing?

- We apologize for this mistake, and this figure is now included.

The Discussion lacks caveats and comparisons of the model with others in the literature. For example, how important is the specific set of assumptions about the friction and the dynamics of the adhesion bonds? The rest of the model is fairly simple linear kinetics, so it would be good to briefly comment on how important the form of equation (6) is.

- We have added some more refs to other relevant studies in the Introduction and compare them to our model.
- The friction and adhesion dynamics which we have in our model, such as in Eq.6 (revised Eq.2), give rise to the stick-slip migration. The stick-slip behavior is important for the dynamics of the branched cell, but it is not the only driving mechanism for oscillations and non-linear dynamics. The non-linear coupling between the polarity-cue distribution and the actin retrograde flow, gives rise to the sharp transitions in the polarity between competing arms, and is the main mechanism driving the out-of-phase oscillations that we found in [26] for a single junction, and we show for highly branched cells in [27]. We have now added this discussion to the Model sections, to emphasize the sources of non-linear dynamics in our model, and how they each contribute to the observed dynamics of the cell.

Minor points

L 39 - ...branching is determined by the network geometry.
L 45 - ... a range of chemical gradient generated by tissue – cells or themselves.
L 54 - …indecision (not in-decision)

- Corrected.

Cell polarization in the model is mentioned on L 195, but it is not explained what this means in terms of the model. What aspect defines that polarization? There is something related to this in Fig S2, but it is not referenced here and that figure is confusing.

- We have added to the main text an expanded explanation of our model, as a new “Theoretical Model” subsection in the “Material and Methods” section. The mechanism by which our model cell spontaneously polarizes is now explained.

If I understood it, the chemokine gradient is linear with the y coordinate of the location, which assumes that the hexagons are entirely permeable to the chemokine. But if the hexagons are impermeable, then the chemokine would be graded along the path lengths. I am wondering if this might help the cells chose the most direct (shortest) paths? Can the authors please comment?

- Calculating the exponential decay of the chemokine along the direct path or along the hexagonal network does not change the profile significantly, it simply makes the chemokine decay faster since the path length is slightly longer than the direct path. It should therefore not change the ability of cells to detect the shorter path, since the chemokine profile is not very different.

Fig 2 shows that total cell length can vary between 3-13 units. Can the authors please comment on how much the real cells stretch in the experiment.

- The cell length changes during the stick-slip migration (beta_0=8, now Fig.3D) is of order of a factor of 2, and similarly for the larger beta_0=9.5 (now Fig.3E), due to the slow-mode behavior. In Fig.6A,B we find in the experiments that the cell length (represented by its area in this data) also changes by a similar factor. We have added a comment regarding this in the main text (Lines 309-312).

The "critical polarization length" L_c is mention on L 242 and 244 but does not seem to appear in the model (appendix S1)? The value itself seems to be dependent on beta as it appears to take different values in Fig 2 C(i) and C(ii).

- The polarization mechanism and its dependence on the cell length is now explained in the new “Theoretical Model, Cell polarization” section.

"Energy" is mentioned once on L 358. It was not clear where this statement came from.

- We removed this mention of energy expenditure. We mention that there is an intermediate regime where the accuracy is still high, while speed is also large enough to provide short arrival times.

FMI (line 319) should be defined somewhere.

- We have added its definition in Lines 380-382.

You use CK-666 and CK666. Please pick one. Searching for either one does not lead to any description of what this inhibitor is doing.

- We have unified the notation to “CK666”. The effects of the drugs are described in Appendix 7 (previous Appendix 5), and the reference to this section has now been added in the main text.

**Reviewer #2:** In this paper the authors applied a mathematical model (that describes cells extending branches to probe their environment) to chemotaxis of neutrophils. In the model, the chemokine enhances local actin activity and thus drives the directed motion. It was found that chemotacting cells exhibit a speed-accuracy trade off, where slow cells can more accurately respond to weak gradients. The model and the two experiments showed similar migration characteristics, indicating general features for neutrophils that are well-described by the model.

- We thank the reviewer for a very supportive report.

General comments

The authors did a very thorough analysis of the model. Furthermore, the model was compared to not one but two experimental set-ups. Showing that the model is well applicable.
Because of the wealth of quantification, figure panels and supporting text and figures, the main message sometimes got lost. I am not sure what to recommend to improve this. The introduction and discussion are relatively short. The impact of this paper could be improved by explaining the broader context and relevance some more. The explanation of the mathematical model can be significantly improved (see specific comments for appendix S1).

- We have indeed improved the manuscript by moving the model description from the Appendix to the “Materials and Methods, Theoretical model” section. We have also expanded the Introduction and Discussion by adding more references to other works, and more detailed discussion of the properties of our model and how they are related to the cells.

Specific comments

Abstract:
Incorrect english / typing mistake in second sentence of abstract “such chemotaxis…”
“of laser-inflicted wound” I think should be “of A laser-inflicted wound”

line 38: if determined → is determined
line 45: generated in by → generated by

- Corrected.

Methods section:
I would personally prefer it if the explanation of the mathematical model and its equations are part of the methods and material section, instead of in the appendix S1. Figure S2 too.
Figure S2 mentions a polarity cue that is not mentioned in Appendix S1. It is as far as I can tell, only firstly mentioned in the main text in Figure S6. I am not sure if this is an additional variable in the model and how it is regulated. Is it c_i(x)?

- We have followed the reviewer’s suggestion and moved both the model description and Fig.S2 into the main text, now in the “Materials and Methods, Theoretical model” section" and Fig.2, respectively.

Results section:

line 219-line 222: can you explain based on the model equations and assumptions why for this range of beta values, the cell starts to stick-slip?

- Stick-slip migration occurs naturally in our model, as was shown in [31]. We have added a new Appendix S2 to demonstrate the value of beta_0 at which stick-slip occurs for the same parameters as in Fig.3 on an infinite 1D line. This critical value of beta_0 is now indicated in new Fig.3B(i) (blue vertical line), and is larger than the value of beta_0 where stick-slip is initiated by the arrival of the cell at the junction. We have added a discussion of this point in Appendix S2 and caption of Fig.3.

Line 297-301: how are Lpath, average v_j and FMI calculated?

- We added a brief definition of these quantities in the main text Lines 379-381.

Figure 3C: the green arrows, should they indicate D(i) to D(iii) instead of E(i) to E(iii)?

- Yes, that was a typo, fixed now.

Line 372-375: Why did the chemokine concentration have to be linear now? And how does the chemokine profile relate to the in-vitro experiment chemical gradient (if known?)

- The gradient of chemokine in-vivo is not known, while in the in-vitro experiments of cells moving in the maze of pillars it is approximately linear [28]. In addition to the simplicity of the linear gradient, we therefore chose to compare the experiments to the simulations in a linear gradient.

Line 416: Can you briefly mention which parameters were changed and why?

- The detailed explanation was given in Appendix S7 (previous Appendix S5), but the reader was not directed to read this section by mistake. This is now corrected.

Line 683: does this refer to the right Figure? I do not see a figure 5E and Supp Figure5E does not seem to relate (unsure).

- By mistake this figure got removed, and it is now added as Fig.S5.

Caption Figure 5 panel C(ii): The two colored regions correspond to the three slow mode
events marked in → The two colored regions correspond to the two slow mode
events marked in

- Corrected.

Discussion section:
What are the model limitations, what can be improved? How do the model results compare to existing literature?

- We have expanded the Discussion to address these very good points, pointing out limitations in our model.

Appendix:

In S1 Appendix, some details are missing for me. The equations are there but the assumptions are not explained well. Without this, it’s hard to gain some intuition and relate it to the biology. I recommend to explain the model more step by step, each term and each parameter and the underlying assumptions. Examples if what is unclear:
What is k in equation 5? Should it be kappa? What role does the term (L-1) play, is L normalized to 1?

- k is the effective cell elasticity. The length in this study is normalized by cell’s rest length, and (L-1) describes the cell’s relative deformation which results in a spring-like restoring force. We expanded this explanation before and after Eq.5.

Line 572: is kappa the same as k?

- kappa is the effective elasticity of adhesion linkers. By mistake it was missing in Eq.8 (revised Eq.4), and now it’s corrected.

I don’t understand what variable describes the direction of the motion of the arm’s leading edge, which apparently is part of equation 8 somehow? Why does the equation 6 and 8 look like this? Is it modeling slip-bond dynamics? What do the two terms in equation 6 describe? What is r in equation 6? What role does n_i play in equation 8? It’s not easy to understand the underlying physics or assumptions in particular in equation 8. Why is the Heaviside function used?

- The direction of the motion of the arm’s leading edge depends on the sign of the dx_i/dt (previous Eq.5, revised Eq.1).
- Eq.6 (revised Eq.2) is the dynamics of the slip-bond adhesions. The first term describes the attachment of the adhesions: new bonds form at a constant rate r and are limited by the fraction of free sites (1−n_i). The second term describes the force-dependent detachment: existing bonds detach with a Bell-type (slip-bond) off-rate that grows exponentially with the load per bond. The net tensile load is the cell-spring tension k(L−1) opposed by protrusive force v_i; this load is shared equally among n_i bonds; f_s is the susceptibility of the adhesion linker to the applied force.
- In previous Eq.8 we used the Heaviside function so that the friction acts as a constant drag when the arm is extending, while it has slip-bond adhesion when the arm is retracting (dx_i/dt<0). We rewrote it in a piecewise form (now Eq.4) to make the underlying physics clearer.

Equation 9: same kind of questions, why this form, explain the dynamics that is described in equation 7.

- In Eq.7 (revised Eq.3), the first term describes the relaxation of the local actin flows toward their steady-state values v_i^* at rate delta. The second term represents stochastic fluctuations in the actin flow velocity, modeled as a Gaussian noise with amplitude sigma. The detailed calculation of v_i^* is now added in S1 Appendix.

slip-bond dynamics: many bonds are now know to behave as catch-bonds too. Do these type of adhesive bonds exhibit catch-bond dynamics in real life and if so, how would it affect the model results?

- The reviewer is correct that catch-bond behavior is important for cell-substrate adhesion. This is mostly important during the extension of protrusions over the surface, allowing the adhesion clusters to grow and mature. We assume here for simplicity that this process is fast and that the substrate offers a constant traction for the actin to convert its polymerization rate into protrusive force that drives the forward extension of the cell edge. Adding a time-scale for this maturation process will slightly slow down the extension of protrusions, but will not qualitatively change the dynamics of the cells.

Line 587 to Line 590: how is the calculation performed? Why is it spatially distributed in the model and not constant? Do these exponential sections mean that the concentration goes exponentially up or down towards the tip?

- We added the detailed calculation of the polarity cue concentration in S1 Appendix.

S3 and S4 Appendix have the same title. Probably a mistake?

- Yes, the title of S4 (revised S5) Appendix is corrected now.

Table S1: “some of the model parameters used in this study” are not all of them included in the table?

- We added a caption to Table S1 explaining that we give here the values of the parameters of the model that we do not change throughout the paper, and hold them constant (almost, some do change in Appendix S7 when simulating the effects of drugs).

I would recommend to add two columns: a short description of the meaning of the parameter, or name of the parameter, and one with the unit of the parameter.

- We added to “S1 Appendix” a Table S-1 which explains the meaning of the parameters and the first equation where they appear. We explained that as in our previous work [26], the model equations are normalized by the inverse focal-adhesion disassembly timescale and by the cell’s rest length scale on one-dimensional tracks [31], so all parameters are dimensionless.

**Reviewer #3:** In this paper the authors formulate a model for chemotaxis of cells having a branched geometry wherein the authors have extended some of their previous work on motility of branched cells to account for bias in cell motility due to chemotaxis. The primary claim of this paper, which has been shown clearly from the mathematical model is that slow-moving cells can chemotax better (Fig 2B, Fig 3C(iii)). This is an extension from their Nature Physics article of 2024, and the modelling is well done. The authors compare the model with chemotaxis of immune cells both in-vitro and in-vivo. This is just what would justify a PLOS computational biology paper. The data are fairly clear.

- We thank the reviewer for the positive and encouraging assessment.

However, it is not clear how the experimental data shown in this paper are in support of the speed-accuracy trade-off hypothesis that is predicted by the model. In fact the only discussion of this hypothesis is provided in the supplementary figure 7 of the paper. Given that this is one of the key novelties of the paper, I would have expected this to be a key figure of the main text and a decent discussion of the same in the main text. However, the only discussion that the authors provide for this is in the form of a single sentence in line 417-418 where the authors write “Comparing the model to the 416 experiments on these drug-treated cells (S7 Fig) gives good qualitative agreement, and points to the WT cells residing in the high-β0 regime of our model”. The authors have not provided any justification for this claim of ‘qualitative agreement’, and as far as I can observe (see below), there isn’t any agreement as far as the speed-accuracy trade-off hypothesis is concerned.

- The reviewer is correct that we presented our results in a misleading way. The clean speed-accuracy tradeoff for a cell on a single junction (Fig.3B(i)) only appears in the limit of very low internal noise in the actin polymerization activity (sigma parameter in our model). As soon as the noise has realistic magnitude, this effect is greatly diminished (Fig. 3B(ii)). We now emphasize this in the discussion of Fig.3.
- For chemotaxis of the branched cells on the network, we find that for low noise and weak signal, there is some remnant of the speed-accuracy tradeoff, as slow cells exhibit straighter trajectories (higher FMI, solid line in Fig.4C(iii)). However, this is again almost fully washed away at higher (more realistic) noise levels, or in the strong signal regime (Fig.4C, S3 Appendix).
- It is therefore not surprising that the FMI of the drug treated (slower) cells are not higher than those of the WT (faster) cells (S7 Appendix). We note that the drug-treated cells are also observed to more often get stuck when migrating in-vivo, presumably as they are weaker and less able to overcome obstacles that the WT cells manage to pass. It is therefore difficult to find consistent changes in the FMI due to the drug treatment in experiments. We note that a recent study found faster cells to have lower chemotactic index [42], where motility was tuned using temperature rather than drug treatment.
- To conclude, our model shows that speed-accuracy over a single junction is a property of our model in the low noise limit. As noise increases this is almost washed away, and is anyway greatly diminished (even at low noise) for branched cells on a network. All this makes the identification of the speed-accuracy relation in experiments challenging.
- However, our model makes other predictions, as we emphasize in the answer to the next point.

In summary, the experimental data presented in this paper does not go far enough to validate verify or validate the key hypothesis presented by the model, meaning that the conclusions (clearly stated in the Abstract) are not yet supported by the data. I therefore cannot recommend publication of this manuscript in the current form in PLOS computational biology. If the authors can obtain data that better supports the model, or justify much more effectively why their data provides such support, the paper would be well suited. Alternative would be to address some of the below points regarding presentation of the model, and publish the model, which is well made and of interest, independent of the experiments in a more specific journal of mathematical biology.

- Following the reviewer’s comments we have now improved the presentation of the comparisons between the model and the experiments. We find it quite compelling that by comparing our model to the experimentally (in-vivo) measured statistics of the short-time single cell polarity and migration response to the chemical signal (Fig.5, S5 Fig, S7 Appendix) we reach the conclusion that the neutrophils correspond to the high beta regime of our model. This conclusion then allows us to make predictions regarding the large scale and long-time behavior of these cells while they perform chemotaxis through complex geometries. Namely, that these cells should exhibit meandering paths (low accuracy) and slow-mode events (where they get transiently stuck in highly elongated shape and indecision). Both of these characteristics are observed in the experimental data (in-vivo and in-vitro), thereby demonstrating the power of our theoretical framework that can allow us to connect and correlate the intricate shape and polarization dynamics of cells to their large-scale migration properties. We hope that the improved presentation conveys these points more clearly.

Specific comments:
- In the experiments, the cells are treated with drugs which either inhibit actin polymerization or myosin-II activity, and the only conclusion that can currently be drawn from these experiments (in-line with previous experiments on cell motility), is that these drugs reduce the overall motility of the cells. None of the results presented in Figure S7 as presented show that the drug-treated cells can chemotax better than the normal ones, and this is something which is also in line with previous experiments. Maybe the authors disagree and can explain more effectively what the data supports the model. But the current experimental data do not support the novel conclusion.

- See our reply to the first general comment of the reviewer regarding the speed-accuracy relation in our model and in the experimental data.

- In lines 207-208, the authors refer to a ‘noise’, it is not clear where this noise comes from, and how it manifests itself in the cell motility. I would expect to see the mathematical equations or some intuitive explanation of this noise term before I see any results where it is varied.

- This refers to the internal noise level in the actin treadmilling flow velocity, as defined in Eq.3 (previous Eq.7). We added now the reference to this equation.

- The authors refer to a ‘slow mode’, as something which occurs if the actin-activity parameter independent of the chemoattractant β_0 increases – this is something which they claim to have found in a previous paper but I struggle make any sense of Figure 2B(i) and I believe I am more expert than most readers. Could the authors offer an intuitive physical or mathematical explanation of the same.

- The slow-mode is characterized by the cell arriving at the junction and getting stuck in indecision for a relatively long time, whereby two elongated and competing arms grow along the new paths. The dynamics is shown in Fig.3E (previous Fig.2C(iii)), where we added now in Fig.3E(iii) a series of snapshots from the simulation to clearly illustrate the shapes of the cell as it gets temporarily “stuck” in the slow-mode event.

- In Figure 2B(i), it could be more appropriate having discrete data points (something like 2C(ii)) because the data presented here is from individual simulations, and not an analytical formula. Furthermore, it is important to mention how many stochastic simulations have been considered while reporting a single data point, and also report the error associated with the data due to stochasticity.

- In the original Fig. 2B, the error bars (gray shaded region) indicated the central range containing 50% of the data around each mean value. We have now modified the figure (now Fig.3) to display discrete data points with mean ± standard error, consistent with the presentation in the multiple-junction case. The number of stochastic simulations and the definition of the error bars are now specified for both cases in the caption.

- In Figure 2B(i), it seems that beyond β_0>11, the cells are unable to make a decision within the simulated time, which could make the definition of P(wrong) ambiguous. My recommendation would be to clarify the definition of these probabilities more carefully.

- For cells that did not make a decision within the maximal simulation time, we assigned the direction of the longest arm at the end of the simulation as the cell’s eventual migration direction, and determined the probabilities based on this. This has now been clarified (Lines 299-301). As shown in Fig.3E (previous Fig.2C), in the low noise limit the longest arm is always the one that eventually decides the direction in which the cell leaves the junction.

- In Figure 4, the authors compare the experimentally observed cell speeds with the y-component of the cell velocity obtained from simulations. I do not understand why it is justified to do that. What this means is that you are comparing one aspect of the experiment with a separate quantity from the model, which appear to be a meaningless comparison. The authors must explicitly justify why they do it or use a fair comparison.

- In our model, cells were restricted to migrate along the edges of the hexagonal lattice, whereas in the experiments the cells move within a more heterogenous background. Therefore, as the cell in the experiments moves towards the wound (chemokine source) and this motion is averaged over many realizations, the migration component that is orthogonal to the wound gets averaged out. Following the reviewer’s comment we now replaced the original panels in Fig.5 with new panels that show the overall cell speed from simulations with respect to LW and BM time, where the hexagonal grids are rotated by 0°, 10°, …, 110° (by symmetry, 120° returns to the original orientation) with respect to the chemokine direction. This procedure averages out the effects of the hexagonal geometry and indeed gives results that are very similar to the experiments.

small errors:
not defining parameters before discussing it (FMI for example),

- We added the definitions of L_path, v_y, and FMI in Lines 379-381.

assuming a reader would have read their previous papers to make sense of the notation that they used previously (eg the stick-slip difference).

- We added a brief description of stick-slip within the context of our model (Lines 100-109).

Please focus on the presentation of the mathematical model to make it clearer and revise the presentation.

- We have moved the whole mathematical model description into the main text with the associated text, so this should be clearer now.
