# Supplementary material for: Modelling chemotaxis of branched cells in complex environments provides insights into immune cell navigation
Source: PLoS Comput Biol. 2026 Feb 3;22(2):e1013934. doi: 10.1371/journal.pcbi.1013934 (PMC12880755; doi:10.1371/journal.pcbi.1013934)
Supplement: Attachment — Submitted filename: ResponseToReviewers_2ndRound.docx [file pcbi.1013934.s016.docx]

**Reviewers' comments:**

Reviewer's Responses to Questions

Reviewer #1: The authors have addressed most of the technical issues in the paper, so the presentation is improved. There are some typos, (including chamotaxis, chemotxis).
That said, my overall impression is that (1) The branching cells model was motivated by experiments (Fig 1, Fig 5A, Fig 6), though the model predictions are not closely compared to those experiments. (2) the model is somewhat arbitrary description of generic cell motility, so may have limited applicability beyond this system; if it agrees well with the experiments, this should have been more strongly demonstrated with various statistical analyses. (3) The authors draw some conclusion on speed vs accuracy tradeoff with noise, but, while there are a lot of specific detailed results on simulation runs, it is hard to understand what aspect of this model lead to those results.
For these reasons, I find it hard to make a recommendation for this paper.

- We address the points raised by the reviewer as follows:

1) The model predictions are compared to the experiments on several levels: In Fig. 5 we compare the details of how cells respond to the chemokine gradient in the way that they effectively rotate their internal polarization and cytoskeletal protrusive activity, resulting in their reorientation to move towards the chemokine source (the wound in the experiments). Using this comparison, we are able to calibrate that the neutrophils correspond to cells with high value of the activity parameter beta in our model (the meaning of “high beta” or “high activity” for neutrophils is now explained in a new Fig.6).

From this comparison we make two additional predictions: First, that due to the high activity the neutrophils should be susceptible to inaccurate chemotaxis and display meandering trajectories towards the chemokine source. Second, that they should get transiently trapped in slow-mode events, which are characterized by the cell extending two long protrusions (elongating the total cell length), whereby the cell is stuck until one of these protrusions is retracted and the cell continues in the direction of the winning one. Both predictions are observed by the neutrophils as shown in Fig.7.

2) The model was originally developed to describe in a very general and simple manner the actin-based migration and the relation between the protrusive activity at the cell tips and the internal polarization of the cell [the Cell paper listed below]. This results in a very powerful, yet simple model that predicted a Universal Coupling between Cell Speed and Cell Persistence (UCSP). This model has since been successfully extended and used to describe cell migration modes in one-dimension and when cells move across junctions and over networks, for different cell types. We therefore view this model as a generic and useful model for describing branched-cell migration, which we are here extending to tackle the problem of chemotaxis.

Maiuri, Paolo, et al. "Actin flows mediate a universal coupling between cell speed and cell persistence." *Cell* 161.2 (2015): 374-386.

Lavi, Ido, et al. "Deterministic patterns in cell motility." *Nature Physics* 12.12 (2016): 1146-1152.

Ron, Jonathan E., et al. "One-dimensional cell motility patterns." *Physical review research* 2.3 (2020): 033237.

Mukherjee, Apratim, et al. "Actin filaments couple the protrusive tips to the nucleus through the I‐BAR domain protein IRSp53 during the migration of cells on 1D fibers." *Advanced science* 10.7 (2023): 2207368.

Ron, Jonathan E., et al. "Polarization and motility of one-dimensional multi-cellular trains." *Biophysical journal* 122.23 (2023): 4598-4613.

Ron, Jonathan E., et al. "Emergent seesaw oscillations during cellular directional decision-making." *Nature Physics* 20.3 (2024): 501-511.

Liu, Jiayi, et al. "Trade-off between branching and polarity controls decision-making during cell migration." *Science Advances* 12.1 (2026): eads2734.

3) We clarified the text and emphasized the main results that the model provides with respect to the speed-accuracy relation:

The speed-accuracy relation, even on a single junction, arises in our model due to the slower cells having to spend longer time over the junction which allows the chemotactic bias to affect them for a longer period. In addition, slower cells have weaker intrinsic polarity, and are less persistent, so more easily respond to the external chemotactic bias. We added these sentences to clarify the origin of the speed-accuracy relation in our model in line 341-345.

On the network, faster, higher activity cells have lower directionality up the chemokine gradient, which is shown in Fig.4 for the case of low noise. As the noise increases this relation weakens, as is shown in Fig.S5. We added to the Discussion section a few more sentences about the speed-accuracy tradeoff predicted by our model, and note that it was also recently obtained in a different theoretical model [25] which we cite and discuss.

Reviewer #2: The article has been improved. The authors have answered most, but not all, of my concerns.

- We thank the reviewer for the positive and constructive report.

In the revised article, the authors mention that the speed-accuracy trade-off is much less pronounced in the presence of realistic levels of noise. I certainly agree with the addition of this important caveat, as indicated by one of the other reviewers. But now it makes me wonder what is the added value of this paper if it’s main message (this trade-off) doesn’t actually occur for realistic parameter values.

- The main message of the paper is to present a theoretical framework that describes the chemotaxis of branched cells in complex geometries, which allows us for the first time to study and analyze such phenomena of living cells. We systematically expose the results of this model, first on directional-decision making over a single junction, and then for chemotaxis of branched cells on a hexagonal network. The speed-accuracy tradeoff is one result, which the model provides, which indeed is difficult to directly observe for realistic noise levels (as the model shows).

However, the model allows us to describe the details of how cells respond to the chemokine gradient and how they effectively rotate their internal polarization and cytoskeletal protrusive activity, resulting in their reorientation to move towards the chemokine source (the wound in the experiments). We compare this model description to the experiments in Fig.5. We added a note about our model’s ability to give a mechanistic description for the rotation of cellular polarity in response to chemotactic signal, which was recently shown to play an important role during chemotaxis [42].

Using this comparison, we are able to calibrate that the neutrophils correspond to cells with high value of the activity parameter beta in our model (the meaning of “high beta” or “high activity” for neutrophils is now explained in new Fig.6). From this comparison we make two additional predictions: First, that due to the high activity the neutrophils should be susceptible to inaccurate chemotaxis and display meandering trajectories towards the chemokine source. Second, that they should get transiently trapped in slow-mode events, which are characterized by the cell extending two long protrusions (elongating the total cell length), whereby the cell is stuck until one of these protrusions is retracted and the cell continues in the direction of the winning one. Both predictions are observed by the neutrophils as shown in Fig.7.

I find the discussion section still very short. Here I would expect a discussion on the biological relevance of the speed-accuracy trade-off. What do we learn from this trade-off that was found in the model, even though cells may not actually enter this ball-park of low noise in real life? Or are there certain situations/conditions in which they do?

- Regarding the speed-accuracy tradeoff that the model predicts (and another recently published theoretical model which we cite [25]), we added that it may inspire future experiments designed to test and observe this behavior with different cell types or under different drug treatments.

Perhaps you can relate it to what is mentioned in the introduction in line 62 to line 68 and in line 540 to line 548 in the discussion section.

Line 62-68: I noticed now that this is a very long sentence, please break it up in more sentences. Currently, is also reads as if you discovered in your simulations what are these constraints for efficient neutrophil response “they must be uniformly distributed at high density throughout the tissue, while having mechanisms for enhancing the chemical signals that facilitate efficient recruitment of neutrophils from larger distances.” but it’s more of a hypothesis that extended from your result, right? And not a direct result from your simulations?

- The reviewer is correct, and we simply removed the last part of this very long sentence in the Introduction, which now reads: “Comparing these characteristics predicted by the model to the experiments (both {in vivo} and {in vitro}), we find that neutrophils behave as the fast cells in our model, thereby providing insights into the constraints that determine the optimal properties for efficient neutrophils response.”

Reading about the trade-off existing only in low noise conditions, also made me wonder when would a slow but accurate path/trajectory be beneficial? If cells just need to arrive at, say, a wound, would it matter how much they meander before they arrive, or is only a fast arrival time necessary? If, say, a cell is chasing a bacteria, perhaps less meandering and more accuracy would be more favorable? Could cell migration noise somehow be targeted by some drug to get cells in this high accuracy and low speed regime, or in the high speed low accuracy regime? Perhaps commenting on this is a good addition for the discussion section.

- Motivated by the reviewer, we added to the discussion a few sentences (Line 562-567) proposing scenarios where accuracy may be more important than speed for motile cells performing chemotaxis.

Discussion section:
Did other modeling methods (for instance the theoretical models described in your introduction) exhibit the speed-accuracy trade-off? How do other results of your model compare to the results of those models? Such a discussion on how your work is placed in the mathematical modeling world is still missing.

- We added to the Discussion section a sentence noting that a somewhat similar speed-accuracy trade-off was recently described in another theoretical model [25].

Methods section:
I understand now that slip-bonds dynamics are part of both equation 1 and 2. I do not really understand the different between the two. I understand why it is part of equation 2. But, for equation 1, when the arm is retracting, the friction depends on slip-bond adhesion behavior. This part I do not understand. I would expect an opposing force due to adhesion given by the variable n, and where n depends on slip-bond dynamics. I must be missing something. Can the authors please explain this better in the Methods section?

- The reviewer is correct, the slip bonds do indeed affect the dynamics of the arms when they retract, and the effective friction coefficient \Gamma_i in Eq.1 does depend on the fraction of slip-bonds that are adhered to the surface (n), as is given in Eq.4. We added a sentence after Eq.4 to emphasize this relation.

Line 494. I would expect the explanation and rationale of which parameters you change to simulate the effect of these drugs to be in the main text and not in the Appendix. Also mention the specific drugs CK666 and blebbistatin in the main text.

- We followed the advice of the reviewer and added this information in the main text.

Minor comments:
Second line of abstract. “Further complicated” doesn’t sound like the right term to me. It suggests that chemical signals also complicate cell motion. Then also, the geometrical aspect is not part of the abstract after this sentence.

- We clarified this part of the abstract.

Last sentence of abstract: “at the expense of compromising their” choose one the two

- We deleted this sentence.

line 14: chamotaxis typo
line 91: the heaviside function doesn’t appear in the formulas anymore
Line 116: explain that c is an inhibitory polarity cue
In response to my previous questions you said that “this load is shared equally among n_i bonds”. Please mention this assumption in Line 100-102.
Line 432/433: there is an empty red box in the white space at the left of this paragraph, that I can click and it leads me to page 20
Line 520-527: There is some repetition here
equation S11 in Appendix S1: some exp are italic
Line 495: there is a small extra red box above the red box around Appendix.

- We addressed these points as instructed by the reviewer.

Reviewer #3: I thank the authors for a careful revision of the paper. While I am in agreement with a majority of revisions made by the authors, I have a couple of major concerns which I believe are absolutely crucial to this paper, and must be addressed before I can recommend publication.
(i) The authors mention: “We note that a recent study found faster cells to have lower chemotactic index [42], where motility was tuned using temperature rather than drug treatment.” I checked this reference, this paper does not make this claim at all, in fact the discussion in this paper says this: “cell migration speed increased with temperature (Figure 1F), without significant changes in the chemotactic index (CI) (Figure 1G)”. The data presented in this paper does not provide any clear evidence to show that faster cells do worse chemotaxis, and moreover, the authors of ref 42 do not make any claims about chemotactic index. Therefore, I would strongly recommend removing this part entirely.

- We follow the reviewer’s suggestion and removed this reference, where the observed decrease in the CI for faster cells was not significant enough.

(ii) “we reach the conclusion that the neutrophils correspond to the high beta regime of our model”, this is very vague – you cannot just map one particular cell to a certain parameter without any point of reference (conceptually this is like saying trains have a high speed, but with respect to what?), it would make sense to conclude that neutrophils have a higher beta than some other cell type for example. I believe that the authors should comment only on the qualitative correlation between the model and experiments, and not make any quantitative statements regarding the experiments, because there isn’t any quantitative similarity.

- The reviewer raises here a very important point which we now address and clarify using a new Fig.6. In this figure we explain the scale of the actin activity parameter beta in our model, and how it maps to the measured speeds of three cell types which we used in previous and the present studies (HUVEC, glioma [26] and neutrophils). This figure explains that our conclusion from Fig.5 (and Fig.S11) that neutrophils correspond to beta values of ~10-13 in our model, fits well with our previous identification of HUVEC and glioma cells as having values of beta~6-7 and ~8-10 respectively. These ranges of values nicely correspond to the hierarchy of cell speeds observed for these cell types, with the neutrophils moving faster than the glioma, which are faster than the HUVEC. We refer to this new figure in the text, and we hope this now clarifies the meaning of identifying the neutrophils as having large value of beta in our model.

In the abstract the authors write: “We find that the model captures the details of the subcellular response to the chemokine gradient, as well as the large scale migration, suggesting that the neutrophils behave as fast cells, at the expense of compromising their chemotaxis accuracy, which explains the functionality of these immune cells.” I find this statement in the abstract to be misleading because the authors do not find any proof of the speed accuracy trade-off. If the authors want to stick to the comparison with experiments in this paper, I think they should restrict themselves to commenting only on the fact that the chemotaxis predicted by the model has qualitative similarity with neutrophil migration seen in experiments, thereby providing a mathematical framework to study chemotaxis in branched networks. Statements like a model capturing a detailed cell migration response can only be made if there is experimental proof for the model prediction (speed accuracy tradeoff in this case). Therefore, I would suggest to modify the abstract accordingly.

- We followed the advice of the reviewer and have modified the abstract accordingly.
